# When Transcriptomics and Metabolomics Work Hand in Hand: A Case Study Characterizing Plant CDF Transcription Factors

**DOI:** 10.3390/ht7010007

**Published:** 2018-02-28

**Authors:** Marta-Marina Pérez-Alonso, Víctor Carrasco-Loba, Joaquín Medina, Jesús Vicente-Carbajosa, Stephan Pollmann

**Affiliations:** 1 Centro de Biotecnología y Genómica de Plantas, Universidad Politécnica de Madrid (UPM)—Instituto Nacional de Investigación y Tecnología Agraria y Alimentaria (INIA), 28223 Pozuelo de Alarcón (Madrid), Spain; martamarina.perez@upm.es (M.-M.P.-A.); victor.carrasco@upm.es (V.C.-L.); jmedina.alcazar@gmail.com (J.M.); jesus.vicente@upm.es (J.V.-C.); 2 Escuela Técnica Superior de Ingeniería Agronómica, Alimentaria y de Biosistemas, Universidad Politécnica de Madrid, 28040 Madrid, Spain

**Keywords:** secondary metabolites, metabolomics, systems biology, plant biology

## Abstract

Over the last three decades, novel “omics” platform technologies for the sequencing of DNA and complementary DNA (cDNA) (RNA-Seq), as well as for the analysis of proteins and metabolites by mass spectrometry, have become more and more available and increasingly found their way into general laboratory life. With this, the ability to generate highly multivariate datasets on the biological systems of choice has increased tremendously. However, the processing and, perhaps even more importantly, the integration of “omics” datasets still remains a bottleneck, although considerable computational and algorithmic advances have been made in recent years. In this mini-review, we use a number of recent “multi-omics” approaches realized in our laboratories as a common theme to discuss possible pitfalls of applying “omics” approaches and to highlight some useful tools for data integration and visualization in the form of an exemplified case study. In the selected example, we used a combination of transcriptomics and metabolomics alongside phenotypic analyses to functionally characterize a small number of Cycling Dof Transcription Factors (CDFs). It has to be remarked that, even though this approach is broadly used, the given workflow is only one of plenty possible ways to characterize target proteins.

## 1. Introduction

The acquisition of highly multivariate “omics” datasets has evolved into an integral component of modern plant biology. Nowadays, the functional characterization of target proteins can hardly be imagined without employing transcriptomics, proteomics, and/or metabolomics approaches. Transcription factors (TFs) represent one particular interesting class of proteins, as they constitute one of the broadest functional protein classes in eukaryotes. For the reference dicot *Arabidopsis thaliana*, it is estimated that about 9–10% of the protein-coding genes encode for TFs [1]. The functional characterization of TFs is not always straightforward, because they are frequently members of large protein families, such as the R2R3-type MYB (myeloblastosis) and basic leucine zipper domain (bZIP) TFs with over 100 and 75 members [2,3], respectively, which sometimes share overlapping functions. Hence, reverse genetics approaches coupled with “omics” experiments do not always result in the identification of specific TF functions, as differences appear to be small and can be masked by functional cooperativity among family members. On the other hand, there are several examples for TFs that mainly function under very particular environmental conditions, adjusting the plant transcriptome to changes in prevailing conditions, such as temperature [4], light availability [5], drought, or pathogen attack [6,7]. With respect to this functional specification, it is comprehensible that the function of a given TF might remain undetected, even though functional null mutants are available. Moreover, the post-translational regulation of TF abundance and action further complicates their characterization, because many plant hormone-based regulatory networks depend on shared perception mechanisms, employing the proteasome 26S machinery to degrade transcriptional repressors [8,9]. Intriguingly, the perception and signal transduction systems of a number of those regulatory circuits also share common basic protein elements, which in turn explains the pleiotropic phenotypes of some of the corresponding mutants [10].

For the given reasons, it becomes clear that the scientific trend goes largely in the direction of a combination of comprehensive reverse genetics studies with available “omics” technologies. Such approaches are likely to provide conclusive evidence of the biological function of studied plant target proteins, and here for TFs in particular. Nevertheless, this tendency brings up the question of how the datasets can be integrated to understand and interpret the results on a systems level. Recent research has provided strong evidence that important insight can be drawn from the integration of the different “omics” levels [11,12]. However, it should not be ignored that the correlations between the different regimes (e.g., the transcriptomics, proteomics, or metabolomics levels) are not always linear and directly applicable [13,14], which significantly hampers the integration of the data.

The aim of this review article is to report on a representative “omics” case study that has been conducted in our laboratories over the last couple of years, combining genetics with transcriptomics and untargeted, as well as targeted, metabolomics [15,16,17]. Obviously, this mini-review has neither the claim to be fully comprehensive nor to represent the exclusive way to connect different “omics” technologies. Especially in the case of TF characterization, other highly valuable high-throughput methods such as chromatin immunoprecipitation sequencing (ChIP-Seq) [18], DNA affinity purification sequencing (DAP-Seq) [19], or protein-binding microarrays [20] have to be mentioned. Nevertheless, in its experimental design, the work used as a guideline for this mini-review is very similar to a multitude of other studies published over the past years, e.g., by Morant and co-workers [21] or Caldana et al. [22]. Hence, we think that it is a good basis for this work. Along the way, possible pitfalls will be discussed, and a number of useful tools for data integration and visualization will be presented.

## 2. Genome-Wide Expression Studies

The objective of the underlying studies was the functional characterization of a small number of plant-specific DNA binding with One Finger (DOF) proteins that contain a highly conserved DNA-binding domain composed of 52 amino acid residues that fold into a C_2_–C_2_ zinc finger structure associated with a basic region that specifically binds to *cis* regulatory elements containing a common 5′-T/AAAG-3′ core motif [23,24]. The *Arabidopsis* and tomato genomes contain a total of 36 and 34 DOF TF proteins, respectively, that have been associated with a wealth of physiological processes, such as for instance seed maturation, germination, and hormone signaling [24,25]. Based on phylogenetic studies, the DOF TFs can be subdivided into four clusters of orthologous gene subfamilies, referred to as clade A to D [26]. Clade D, both in *Arabidopsis* and tomato, contains a group of five DOF factors whose transcript levels oscillate under constant light conditions. Due to this property, they are termed *Cycling Dof Factors* (*CDF1–5*) [27,28]. Besides their role in controlling photoperiodic flowering [29], it has been reported that CDF TFs are differentially induced upon osmotic, salt, heat, and low-temperature stress, indicating that they may also contribute to the responses to those types of stresses in overlapping signal transduction pathways [30,31].

In our studies, we focused on the investigation of the role of CDF1 and CDF3 from tomato [15,17] and CDF3 from *A. thaliana* [16] in biological processes in vivo. To do so, we took a gain-of-function approach, which was substantiated by the analysis of knockout mutants if applicable. Alongside a thorough phenotypic analysis, the initial step in the elucidation of CDF functions has been a comprehensive transcriptomics assessment of CDF overexpressing plant lines (CDFoe) under various different growth conditions, including cold, drought, osmotic, and salt stress. The CDFoe lines displayed enhanced drought and low temperature resistance in *Arabidopsis*, and some of the gain-of-function lines also exhibited an enhanced photosynthetic capacity. With the aim of gaining deeper insight into the underlying molecular mechanisms triggered by the overexpression of the CDF genes, comprehensive transcriptomic analyses were performed either by DNA microarrays (Affymetrix ATH1) or RNA-Seq. The obtained microarray data were edited according to the MIAME standards [32] and made available to the community along with the corresponding publications. In case of RNA-Seq data, there is still no general guideline of the minimum requirements that should be applied as a standard for the recording and reporting of next generation sequencing-based gene expression data published [33]. This makes the exchange of RNA-Seq and other massive sequencing data a delicate issue. However, there are a number of attempts of different consortia to change this prevailing lack of information. As an example, the Functional Genomics Data Society (FGED; [34]) has published a MINSEQE guideline on their webpage. In addition, the ENCODE (Encyclopedia of DNA Elements) consortium has released experimental data standards and processing pipelines for numerous different next generation sequencing platform technologies [35]. Ideally, a general and easy-to-use way will be established that facilitates standardized data sharing in the near future. The FAIR data initiative, with its FAIR data principles, offers an extremely interesting starting point in this context [36,37] that deserves the fullest support of the community. In any case, by sticking closely to the proposed MINSEQE guideline, the obtained RNA-Seq data were also made available to the public.

**Figure 1:**
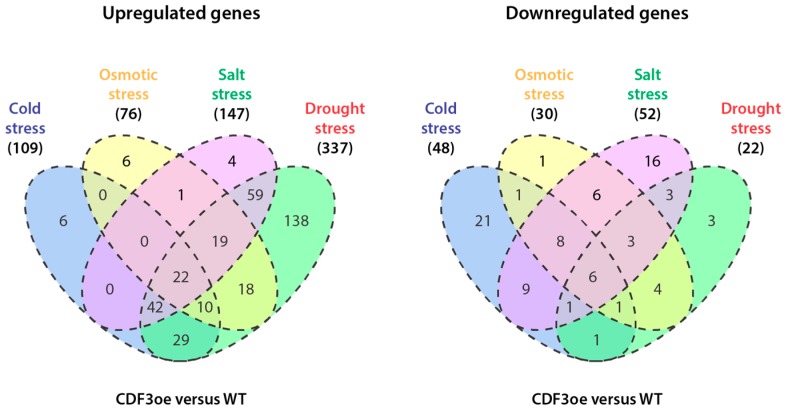
Grouping of differentially expressed genes in CDF3 overexpressing (CDF3oe) lines relative to wild-type *Arabidopsis*. The Venn diagrams show the overlap of up- and down-regulated genes expressed in the CDF3 overexpression line compared to wild type plants in response to different types of abiotic stresses. WT: wild type. Adapted from [16].

Applying an arbitrary threshold of a fold change of >1.5 and a *p*-value ≤ 0.05, we identified around 600 differentially expressed genes (DEGs) in CDF3oe plants. Of these DEGs, around two-thirds were upregulated, while 122 DEGs exhibited a transcriptional repression. In silico analysis of the DEGs and their subsequent classification according to their participation in responses to cold, osmotic, salt, and drought stresses, shown in Figure 1, was carried out using the e-Northern Expression Browser tool [38].

Subsequently a Gene Ontology analysis, using the agriGO Gene Ontology Analysis Toolkit [39,40], and the REVIGO Gene Ontology visualization tool [41], was conducted. Although the GO analysis revealed a clear enrichment of terms related to abiotic stress responses, no conclusive evidence for the specific induction or repression of entire pathways could be unveiled by tools like MAPMAN [42] or AraCyc [43]. Hence, it was concluded that phenotypic analyses and the transcriptomics datasets are not sufficient to fully disclose the molecular intricacies that confer enhanced drought resistance to the CDF3oe transgenic plants, and that an additional high-throughput technology, such as metabolomics assays, should be applied. This decision was further supported by a number of observations that highlighted that drought stress induces major changes in the chemical composition of plants [44,45,46].

## 3. Untargeted and Targeted Metabolomics Studies

The expression “metabolomics” was coined at the dawn of the third millennium describing the attempt to analyze all small molecule metabolites (or at least a large number) in a given biological specimen at the same time. In general terms, metabolomics is defined as the comprehensive profiling of small molecules (i.e., chemical substances that present a molecular mass between 50 and 2000 Da) that can be extracted and analyzed from isolated cellular compartments, whole cells, tissues, organs, entire organisms, biofluids, or environmental samples. Obviously, analyzing metabolites with highly diverse physico-chemical properties constitutes a tremendous difficulty that already begins with the putatively most trivial step, the extraction of the samples. Due to its amazing complexity, metabolomics is most probably more prone to technical problems and limitations than other “omics” regimes and, therefore, needs more commitment and practical knowledge in the laboratory (reviewed in [47]). In reality, this novel ”omics” platform technology is nothing else than well-known analytical chemistry, which of course has a far-reaching history. However, the advent of metabolomics approaches has been substantially fostered by major advances in instrument technology. Most importantly, the robustness, velocity, resolution, sensitivity, and mass accuracy of modern mass spectrometers, as well as their coupling to gas and liquid chromatographic separation systems, has considerably improved over the last fifteen to twenty years.

Due to optimized sample separation techniques, the complexity of the analyte mixture that enters the detector for simultaneous analysis has been notably decreased, which in turn facilitates the detection of over 10,000 molecular features in untargeted metabolomics approaches (“shotgun” metabolomics) in a single biological sample [48,49]. Currently, not feature detection, but rather the association of the detected peaks to specific chemical structures, represents the analytic bottleneck. As a rule of thumb, one has to be aware that in the best of such approaches only around one third of the identified peaks can be attributed to defined chemical structures, and even more importantly, the association of peaks/ion masses to small chemical substances can consume several weeks of work. Moreover, as with the reporting of massive sequencing data, there is no fully established guideline for the deposition and presentation of metabolomics data, although a number of publications give valuable advice on good laboratory practices in this regard [50,51,52,53]. Since the formation of the Metabolomics Standards Initiative (MSI) in 2005, a number of metabolomics data repositories, such as MetaboLights, Metabolomics Workbench, Metabolomics Repository Bordeaux (MeRy-B), or the Metabolic Phenotype Database (MetaPhen) have been developed [54,55,56,57,58]. Those databases represent a rich resource of data, in particular for metadata studies. In addition to those data repositories, just recently, a number of initiatives have been implemented to establish a comprehensive and standardized computational infrastructure that supports data processing and analysis for metabolomics datasets, i.e., PhenoMeNal [59] and MetaboFlow [60].

**Figure 2:**
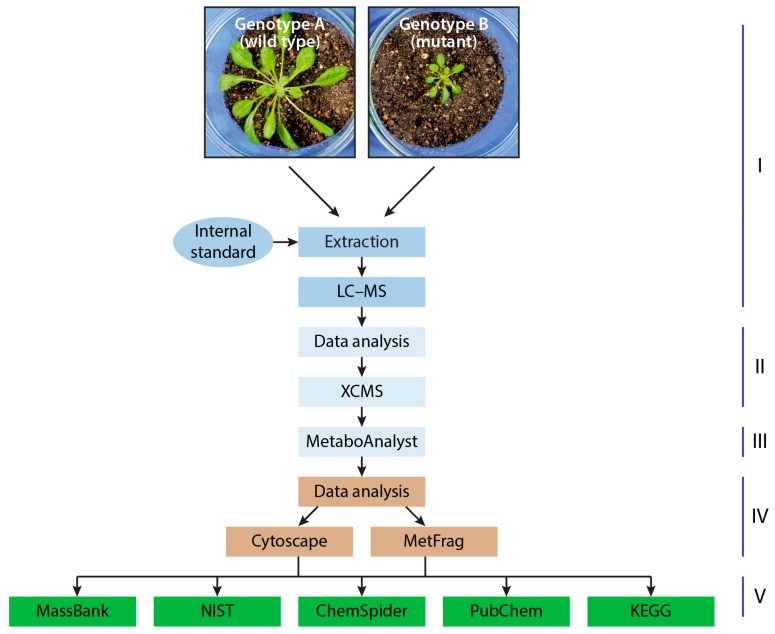
Experimental workflow from sample extraction to the identification of small molecules. (**I**) Sample processing and mass spectrometric analysis; (**II**) data extraction (Data Analysis v4.0, BRUKER Daltonics, Bremen, Germany), peak identification, and alignment (XCMS [64]); (**III**) advanced statistics (MetaboAnalyst v3.6 [65,66]); (**IV**) fragment ion mass extraction (tandem mass spectrometry; MS/MS) and molecular formula prediction (Data Analysis v4.0, BRUKER Daltonics, Bremen, Germany), and molecular feature interpretation (Cytoscape v3.6 [67] & MetFrag [68]); (**V**) association of molecular feature with corresponding small molecules (Database query and literature searches). LC-MS: Liquid chromatography–mass spectrometry; ChemSpider [69]; KEGG: Kyoto Encyclopedia of Genes and Genomes [70]; MassBank [62]; NIST: National Institute of Standards and Technology Mass Spectral Library 2011 (NIST11); PubChem [71].

Despite its complexity, untargeted metabolomics represents a commonly employed technique for comparing biological conditions, for example wild-type plants with knockout or overexpression lines and treated with non-treated control plants, respectively. As such, shotgun metabolomics is an ideal discovery tool for the detection of metabolic changes in response to manipulations of the biological control system. A general workflow for untargeted metabolomics analyses is depicted in Figure 2.

Over recent years, the number of freely and commercially available mass spectrometry (MS) and tandem mass spectrometry (MS/MS) databases has significantly increased, which has gradually simplified the identification of small molecules by library enquiry [61]. To give an example, the MassBank online repository is a large online database that comprises thousands of mass spectra from different instruments and about thirty contributing laboratories [62]. The MassBank database was originally launched in Japan in 2006 and is among the most popular community resources for mass spectral data. Another popular database, particularly for the analysis of gas chromatography–mass spectrometry (GC-MS) data, is the Golm Metabolome database (GMD) [63]. As with the MassBank database, the GMD is publicly available and free of charge.

Coming back to the analysis of the CDF3 overexpressing lines, we started with a shotgun metabolomics approach by comparing the overexpressor plants with wild-type control plants raised under identical, standardized growth conditions. As with the transcriptomics approach, 10 day-old seedlings were used for metabolomics assessment. Obviously, this impedes both organ- and cell-specific analysis, yet it represents a common practice in plant metabolomics. Due to the physical detection limits of current mass spectrometers, it is necessary to start the metabolite extraction with at least 10–20 mg of plant material in order to obtain reliable and robust datasets. However, over the past decades, an enormous amount of biochemical data has been collected, which has culminated in the generation of comprehensive metabolic pathway databases, such AraCyc [43] and the Kyoto Encyclopedia of Genes and Genomes (KEGG) [70]. These databases do not only contain plain metabolic data, but also valuable information on the cellular location of pathways. Together with the information on the employed extraction procedure, this allows an association of compounds with compartments and pathways. It has to be remarked that, e.g., organ-specific differences, like in case of jasmonate-mediated induction of auxin biosynthesis [72] or trans-organ metabolite gradients [73], are not detected when using whole seedlings or plants. This type of analysis is, however, not the claim of a shotgun approach, which is more directed towards the identification of global changes, while targeted approaches are much better suited to more detailed, cell type-specific studies.

**Figure 3:**
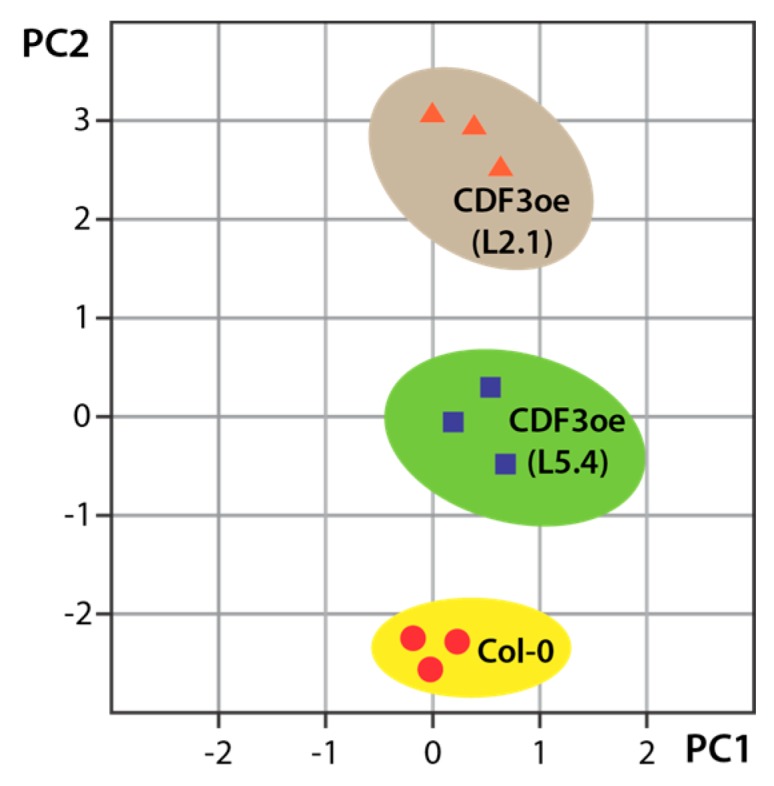
Principal component analysis (PCA) of recorded, untargeted metabolic profiles. The projection plot is shown for principal component 1 (PC1, 55.3% explained variance) and principal component 2 (PC2, 28% explained variance). The unbiased distinct grouping supports the notion of metabolic differences among the explored genotypes (wild-type control, Col-0; CDF3oe lines L2.1 and L5.4), with CDF3oe line L2.1 showing a more pronounced difference to the wild type.

After the extraction of molecular features using the find molecular feature (FMF) algorithm included in the vendor software (Data Analysis v4.0, BRUKER Daltonics, Bremen, Germany), a compound-based principal component analysis was carried out [74]. The principal component analysis (PCA) was conducted using the Profile Analysis v2.0 software (BRUKER Daltonics, Bremen, Germany) and the XCMS [64] and MetaboAnalyst [65,66] online analysis tools. The analysis revealed considerable metabolic differences between gain-of-function CDF3 lines and control plants (Col-0) (Figure 3), which is reflected by the groupation of the three sample sets.

As can be taken from Figure 3, it was possible to detect a difference between the two studied overexpression lines. This can likely be attributed to the different expression levels of the introduced transgene in the two lines. Although the construct for the generation of the transgenic plants was the same, the insertion locus of the transfer DNA (T-DNA) cannot be controlled. Such location-dependent effects can eventually bias the transcription rate of the 35S-driven target gene [75].

**Figure 4:**
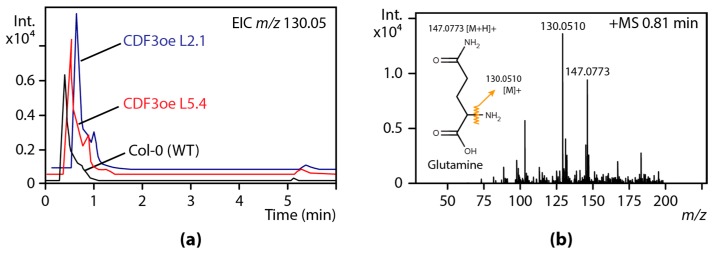
Metabolic analysis of CDF3oe lines in comparison with wild-type *Arabidopsis*. (**a**) The extracted ion chromatograms (EICs) for the mass *m*/*z* 130.05 at 0.81 min reveal elevated intensities of this mass in CDF3oe plants compared to the wild-type control (WT); (**b**) the accurate mass of the parent ion and its isotopic pattern led to the unambiguous identification of l-glutamine.

After the identification of a number of differentially abundant molecular features among the datasets, we next strived for small molecule identification. On the bases of the accurate *m*/*z* values for the pseudomolecular ions, [M + H]^+^, we employed the deviation of both the mass positions and the intensity ratios of the isotopic peaks to predict the molecular formulae of corresponding compounds using the SigmaFit approach [76]. The analysis led to the identification of a number of differentially abundant amino acids and amino acid conjugates in the samples. An example of the differential abundance of glutamine is given in Figure 4.

Although it was possible to identify a list of differentially abundant compounds among the tested genotypes, the overall outcome of the experiment was not fully satisfying. The majority of the identified compounds were either amino acids and amino acid derivatives, respectively, or sugars, hence, the relatively small and, more importantly, charged compounds, which were not considerably retained on the routinely utilized C18 ultra-high performance liquid chromatography (UHPLC) column. This means that most of the compounds eluted within a very short period of time at the very beginning of the reverse-phase chromatographic separation. To tackle this kind of problem, it would have been possible to change the chromatographic system, e.g., by applying a hydrophilic interaction chromatography (HILIC) column. However, in the described cases, it was decided to go for a targeted metabolomics approach, quantifying all proteinogenic and some non-proteinogenic amino acids alongside a number of selected mono- and disaccharides and some small acids, such as lactic, malic, and citric acid by means of GC-MS/MS.

In contrast to shotgun metabolomics, targeted metabolomics focuses on the analysis of a defined number of known metabolites in clusters with comparable chemical properties and structures, such as the above-mentioned amino acids, organic acids, and saccharides. One of the major advantages of targeted metabolomics resides in the possibility of conducting quantitative analyses through the utilization of stable isotope-labeled internal standards containing either ^2^H, ^15^N, or ^13^C labeled atoms, replacing some of the normal isotopes [77,78]. By spiking the samples with a cocktail of stable isotope-labeled internal standards at a known concentration, it becomes possible to deduce the quantity of target analytes through the ratio of their peak area to that of the corresponding added labeled standard, a commonly used workflow in analytic chemistry referred to as isotope dilution mass spectrometry (IDMS) [79,80]. This approach, however, is limited to a manageable number of metabolites, because the list of commercially available stable isotope-labeled compounds is by far smaller than the number of typically analyzed metabolites. Apart from elevated price tags for stable isotope-labeled compounds and an increased complexity of the analyte mixture, this makes absolute quantification in ample targeted metabolomics analyses difficult. In practice, absolute quantification of analytes is therefore often realized by adding a single labeled standard per metabolite class. This pseudoquantitative analysis normally comprises accuracy levels that are comparable to those of real quantitative measurements [81]. As targeted metabolomics provides absolute or relative data on the abundance of a pre-selected number of known metabolites, the results can be co-analyzed along with other “omics” data to generate correlation or partial correlation network maps that are based on, e.g., transcriptomics and metabolomics datasets. Due to the already-mentioned, sometimes poor correlation of the datasets, however, the generation of multi-omics-based network maps remains a great challenge [82]. Nevertheless, the network-based visualization of “omics” datasets can be very helpful for developing and communicating the scientific results of a realized study. Obviously, such a, in part, simplified representation of the data, which is inherently prone to misinterpretation, cannot replace a thorough, unbiased statistical data analysis, and should therefore not be considered as the final result of a study, but rather as a useful visual tool for transmitting the main finding to a broader community.

To date, there is a range of different software tools available that facilitate network-based visualization of metabolomics data and, to some extent, multi-omics data. To name just a few, commercial software applications include Omix [83], which is well suited for handling metabolic networks and comes with some modeling capacity, and Ingenuity Pathway Analysis [84], which is strong at linking custom data to pre-defined canonical pathways. Very sophisticated open-source software solutions include Metscape2 [85], which is an add-on to the popular Cytoscape software that allows the entry of data on metabolites, genes, and pathways in order to display them in the context of metabolic networks. In addition, there are platform independent online tools available, such as ProMeTra [86] and Paintomics [87], or MetaMapR [88]; the latter is an association-based analysis and visualization tool that leverages the KEGG and PubChem databases to also integrate unknowns in given analyses.

**Figure 5:**
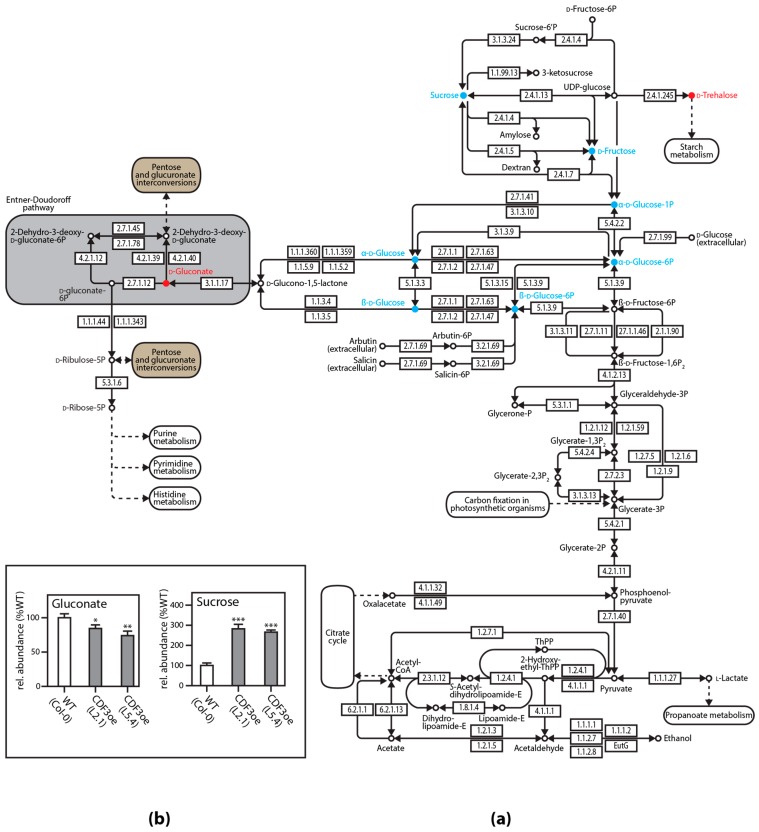
Exemplary results of the targeted metabolomics analysis of CDF3oe plants relative to the wild type. (**a**) Metabolic network map of major changes due to the overexpression of CDF3. According to the obtained data, sucrose metabolism is strongly induced in CDF3oe, which goes at the expense of the pentose phosphate pathway. More abundant metabolites are shown in cyan, and less abundant metabolites are shown in red; (**b**) representation of relative contents of marker compounds for starch and sucrose metabolism (sucrose) and the pentose phosphate pathway (gluconate). CoA: Coenzyme A; E: Enzyme-bound metabolites; EutG: Alcohol dehydrogenase (NP_416948); ThPP: Thiamine pyrophosphate; P: Phosphate; UDP: Uridine diphosphate.

As the final step in the analysis of the CDFoe lines, a targeted metabolomics method was established that permitted the simultaneous quantification of 72 compounds by GC-MS/MS. The performed analysis generated highly interesting results, indicating significant changes in the abundance of, among others, sucrose, γ-aminobutiric acid (GABA), l-proline, l-glutamine, succinate, fumarate, malate, and gluconate. The results have been statistically analysed using univariate and multivariate analyses, which finally led to the construction of the metabolic network map presented in Figure 5. The identified amino acids, and possibly also further amino acid conjugates identified in the untargeted metabolomics approach, are likely the reason for the observed improved stress resistance in CDFoe lines. In particular, proline metabolism is known to affect cellular redox homeostasis, which in turn can help to ensure cell survival [89]. However, other amino acids and, more importantly, sucrose are also discussed for mitigating drought and osmotic stress effects in plants [90,91].

## 4. Conclusions

In summary, it can be noted that single-omics approaches, e.g., microarray analyses, at times reach their limits and do not provide sufficient information to fully describe physiological phenomena, such as enhanced stress tolerance. The picture gets even more complex when pleiotropic growth regulators, such as TFs, are targets of the investigation. Employing a multi-omics approach can considerably help to reveal the biological function of a molecular target. However, it has to be remarked that, at present, only microarray analyses are almost standardized, whereas guidelines for best practices in next generation sequencing and metabolomics are still lagging behind. Nevertheless, as discussed, currently there are already several helpful tools available that make data integration and visualization easier. Eventually, this substantial simplification of data handling is expected to considerably fuel the emergence of multi-omics approaches. Generally speaking, an increased demand mostly translates into improved offers, which promises that more sophisticated bioinformatics tools for the analysis and handling of multi-omics datasets will be elaborated and launched in the near future.
